# Noncontextuality with marginal selectivity in reconstructing mental architectures

**DOI:** 10.3389/fpsyg.2015.00735

**Published:** 2015-06-17

**Authors:** Ru Zhang, Ehtibar N. Dzhafarov

**Affiliations:** Department of Psychological Sciences, Purdue UniversityWest Lafayette, IN, USA

**Keywords:** interaction contrast, mental architectures, noncontextuality, response time, selective influences, series-parallel network

## Abstract

We present a general theory of series-parallel mental architectures with selectively influenced stochastically non-independent components. A mental architecture is a hypothetical network of processes aimed at performing a task, of which we only observe the overall time it takes under variable parameters of the task. It is usually assumed that the network contains several processes selectively influenced by different experimental factors, and then the question is asked as to how these processes are arranged within the network, e.g., whether they are concurrent or sequential. One way of doing this is to consider the distribution functions for the overall processing time and compute certain linear combinations thereof (interaction contrasts). The theory of selective influences in psychology can be viewed as a special application of the interdisciplinary theory of (non)contextuality having its origins and main applications in quantum theory. In particular, lack of contextuality is equivalent to the existence of a “hidden” random entity of which all the random variables in play are functions. Consequently, for any given value of this common random entity, the processing times and their compositions (minima, maxima, or sums) become deterministic quantities. These quantities, in turn, can be treated as random variables with (shifted) Heaviside distribution functions, for which one can easily compute various linear combinations across different treatments, including interaction contrasts. This mathematical fact leads to a simple method, more general than the previously used ones, to investigate and characterize the interaction contrast for different types of series-parallel architectures.

## 1. Introduction

The notion of a network of mental processes with components selectively influenced by different experimental factors was introduced to psychology in Saul Sternberg's (1969) influential paper. Sternberg considered networks of processes *a*, *b*, *c*, … involved in performing a mental task. Denoting their respective durations by *A*, *B*, *C* …, the hypothesis he considered was that the observed response time *T* is *A* + *B* + *C* + …. One cannot test this hypothesis, Sternberg wrote, without assuming that there are some factors, α, β, γ, …, that selectively influence the durations *A*, *B*, *C* …, respectively. Sternberg's analysis was confined to stochastically independent *A*, *B*, *C*, …, and the consequences of the assumptions of seriality and selective influences were tested on the level of the mean response times only.

Subsequent development of these ideas was aimed at the entire distributions of the response times and at a greater diversity and complexity of mental architectures than just series of “stages.” This development prominently includes Townsend (1984, 1990a,b); Schweickert and Townsend (1989); Townsend and Schweickert (1989); Roberts and Sternberg (1993); Townsend and Nozawa (1995); Schweickert et al. (2000), and several other publications, primarily by James Townsend and Richard Schweickert with colleagues. For an overview of these developments see Dzhafarov (2003) and Schweickert et al. (2012). In the present context we should separately mention the development of the ideas of *stochastic ordering* of processing times in Townsend (1984, 1990a) and Townsend and Schweickert (1989); as well as the idea of *marginal selectivity* (Townsend and Schweickert, 1989).

The notion of selective influences also underwent a significant development, having been generalized from stochastically independent random variables to arbitrarily interdependent ones (Dzhafarov, 2003; Dzhafarov and Gluhovsky, 2006; Kujala and Dzhafarov, 2008; Dzhafarov and Kujala, 2010, in press). The essence of the development is easy to understand using two random variables (e.g., process durations) *A*, *B* selectively influenced by two respective factors α, β. In Dzhafarov's (2003) notation, this is written (*A*, *B*) ↫ (α, β). According to the definition given in Dzhafarov (2003), this means that there are functions *f* and *g* and a random variable *R* (a *common source of randomness*) such that *f*(α, *R*) = *A* and *g*(β, *R*) = *B*. If such a choice of (*f*, *g*, *R*) exists, it is not unique. For instance, *R* can always be chosen to have any distribution that is absolutely continuous with respect to the usual Borel measure on the real line (e.g., a standard uniform, or standard normal distribution, see Dzhafarov and Gluhovsky, 2006). However, a triple (*f*, *g*, *R*) need not exist. It does not exist, e.g., if marginal selectivity (Townsend and Schweickert, 1989) is violated, i.e., if the distribution of, say, *A* at a given value of α changes in response to changing β. But marginal selectivity is not sufficient for the existence of a triple (*f*, *g*, *R*). Let, e.g., α and β be binary factors, with values 1, 2 each, and let the correlation ρ between *A* and *B* for a treatment (α, β) be denoted ρ_αβ_. Then the triple in question does not exist if the correlations violated the “cosphericity test” (Kujala and Dzhafarov, 2008), also known in quantum mechanics as Landau's inequality (Landau, 1988):

(1) $$\left|\rho_{11}\rho_{12}-\rho_{21}\rho_{22}\right|\leq {\bar{\rho }}_{11}{\bar{\rho }}_{12}+{\bar{\rho }}_{21}{\bar{\rho }}_{22},$$

where ${\bar{\rho }}_{\alpha \beta }=\sqrt{1-\rho_{\alpha \beta }^{2}}$. There are many other known conditions that must be satisfied for the existence of a triple (*f*, *g*, *R*) when marginal selectivity is satisfied (Dzhafarov and Kujala, 2010, 2012a,b, 2013, 2014a).

The allusion to quantum mechanics is not accidental: as shown in Dzhafarov and Kujala (2012a,b), the theory of selective influences in psychology can be viewed as a special application of the theory of (non)contextuality. This theory is interdisciplinary (Khrennikov, 2009; Dzhafarov and Kujala, 2014b,c,d), but its origins are in quantum theory, dating from Kochen and Specker (1967) and Bell's (1964, 1966) celebrated work. For the modern state of the theory see Dzhafarov et al. (in press). A simplified account of the (non)contextuality analysis of the example given above is as follows. One labels each random variable in play *contextually*, i.e., by what property is being measured/recorded under what treatment (context):

(2) $$\begin{array}{l} {\left(\underset{\text{property: what is measured}}{\underset{︸}{A_{\text{value of }\alpha }}}\right)}^{\underset{\text{context: under what treatement}}{\underset{︸}{\left(\text{value of α},\text{value of }\beta \right)}}},\\ {\left(\underset{\text{property: what is measured}}{\underset{︸}{B_{\text{value of }\beta }}}\right)}^{\underset{\text{context: under what treatement}}{\underset{︸}{\left(\text{value of }\alpha ,\text{value of }\beta \right)}}}. \end{array}$$

The notation here is, of course, redundant, because the context and property identifiers overlap, but we need now to emphasize the logic rather than achieve notational convenience. Once the labeling is done, one looks at all possible joint distributions imposable on all these random variables, for all properties and all treatments. A system is noncontextual if there exists such a joint distributions in which any two random variables that represent the same property (“what is measured”) are equal with probability 1. The latter is possible only if the random variables representing the same property always have the same distribution: in our case

(3) $${\left(A_{\alpha }\right)}^{\left(\alpha ,\beta \right)}\sim {\left(A_{\alpha }\right)}^{\left(\alpha ,\beta^{\prime }\right)},{\left(B_{\beta }\right)}^{\left(\alpha ,\beta \right)}\sim {\left(B_{\beta }\right)}^{\left(\alpha^{\prime },\beta \right)}$$

for any values α, β, α′, β′ of the two factors. This is called consistent connectedness (Dzhafarov et al., in press), and in physics is known under a variety of names, including (in certain paradigms) “no-signaling condition” (Popescu and Rohrlich, 1994; Cereceda, 2000; Masanes et al., 2006). In psychology, this is marginal selectivity. The definition of noncontextuality just given is not the most general one, as the notion of contextuality can be extended to inconsistently connected (violating marginal selectivity) systems (Dzhafarov et al., in press), but we do not need this generality in this paper. What is important for us here is that the existence of a joint distribution mentioned in our definition is equivalent to the existence of a random variable *R* and the functions *f*, *g* mentioned in the introductory paragraph.

It is easy to show (Dzhafarov, 2003) that the existence of a triple (*f*, *g*, *R*) for given joint distributions of (*A*, *B*) under different treatments (α, β) is equivalent to the existence of a quintuple (*f*′, *g*′, *S*, *S*_*A*_, *S*_*B*_), where *S*, *S*_*A*_, *S*_*B*_ are random variables, such that *f*′(α, *S*, *S*_*A*_) = *A* and *g*′(β, *S*, *S*_*B*_) = *B*. In such a representation, one can speak of a common source of randomness *S* and specific sources of randomness *S*_*A*_, *S*_*B*_. In Dzhafarov et al. (2004) this representation was used to investigate different series-parallel arrangements of the hypothetical durations *A* and *B*. The reason this representation has been considered convenient is that if one fixes the value *S* = *s*, then *f*′(α, *s*, *S*_*A*_) = *A*_*c*_ and *g*′(β, *s*, *S*_*B*_) = *B*_*c*_ are stochastically independent random variables. One can therefore use theorems proved for stochastically independent selectively influenced components (Schweickert et al., 2000) to obtain a general result by averaging across possible values of *s*. For instance, let α, β be binary factors (with values 1, 2 each), and let us assume that the observed duration *T*_αβ_ is min(*A*_α_, *B*_β_) for every treatment (α, β). Then *T*_αβ*s*_ = min(*A*_α*s*_, *B*_β*s*_) for every value *S* = *s*, and it is known that, for the independent *A*_α*s*_, *B*_β*s*_ (satisfying a prolongation condition, as explained below),

(4) $$\mathrm{Pr}\left(T_{11s}\leq t\right)-\mathrm{Pr}\left(T_{12s}\leq t\right)-\mathrm{Pr}\left(T_{21s}\leq t\right)+\mathrm{Pr}\left(T_{22s}\leq t\right)\leq 0.$$

Since this should be true for every value *S* = *s*, then it should also be true that

(5) $$\begin{array}{l} C\left(t\right)=\mathrm{Pr}\left(T_{11}\leq t\right)-\mathrm{Pr}\left(T_{12}\leq t\right)-\mathrm{Pr}\left(T_{21}\leq t\right)\\ \;+\mathrm{Pr}\left(T_{22}\leq t\right)\leq 0. \end{array}$$

This follows from the fact that

(6) $$\mathrm{Pr}\left(T_{\alpha \beta }\leq t\right)=\int \mathrm{Pr}\left(T_{\alpha \beta s}\leq t\right)\text{dm}\left(s\right),$$

where m(*s*) is the probability measure for *S*, and the integration is over the space of all possible *s*. The linear combination *C*(*t*) in (5) is called the *interaction contrast of distributions functions*.

The Prolongation Assumption used in Dzhafarov et al. (2004), and derived from Townsend (1984, 1990a) and Townsend and Schweickert (1989), is that, for every *S* = *s*,

(7) $$\mathrm{Pr}\left(A_{1s}\leq t\right)\geq \mathrm{Pr}\left(A_{2s}\leq t\right),\;\mathrm{Pr}\left(B_{1s}\leq t\right)\geq \mathrm{Pr}\left(B_{2s}\leq t\right).$$

For this particular architecture, *T* = min(*A*, *B*), this is the only assumption needed. To prove analogous results for more complex mental architectures, however, one needs additional assumptions, such as the existence of density functions for *A*_α*s*_, *B*_β*s*_ at every *s*, and even certain ordering of these density functions in some vicinity [0, τ].

The same results, however, can be obtained without these additional assumptions, if one adopts the other, equivalent definition of selective influences: *f*(α, *R*) = *A* and *g*(β, *R*) = *B*, for some triple (*f*, *g*, *R*). If such a representation exists, then

(8) $$a_{\alpha r}=f\left(\alpha ,r\right),b_{\beta r}=g\left(\beta ,r\right)$$

are deterministic quantities (real numbers), for every value *R* = *r*. Any real number *x* in turn can be viewed as a random variable whose distribution function is a shifted Heaviside function

(9) $$h\left(t-x\right)=\{\begin{array}{c} 0,\;if\;t<x,\\ 1,\;if\;t\geq x. \end{array}$$

In particular, the quantity *t*_αβ*r*_ = min(*a*_α*r*_, *b*_β*r*_) for the simple architecture *T* = min(*A*, *B*) considered above is distributed according to

(10) $$h\left(t-t_{\alpha \beta r}\right)=h_{\alpha \beta r}\left(t\right).$$

Let us see how inequality (5) can be derived using these observations.

We first formulate the *(conditional) Prolongation Assumption*, a deterministic version of (7): the assumption is that *f*, *g*, *R* can be so chosen that for every *R* = *r*,

(11) $$a_{1r}\leq a_{2r},\;b_{1r}\leq b_{2r}.$$

Without loss of generality, we can also assume, for any given *r*,

(12) $$a_{1r}\leq b_{1r}$$

(if not, rename *a* into *b* and vice versa).

*Remark* 1.1. The Prolongation Assumption clearly implies (7). Conversely, if (7) holds, one can always find functions *f*, *g*, *R* for which the Prolongation Assumption holds in the form above. For instance, one can choose *R* = (*S*, *S*_*A*_, *S*_*B*_), take *S*_*A*_ and *S*_*B*_ to be uniformly distributed between 0 and 1, and choose *f*(α, …), *g*(β, …) to be the quantile functions for the hypothetical distributions of *A* and *B* at the corresponding factor levels.

We next form the conditional (i.e., conditioned on *R* = *r*) interaction contrast

(13) $$c_{r}\left(t\right)=h_{11r}\left(t\right)-h_{12r}\left(t\right)-h_{21r}\left(t\right)+h_{22r}\left(t\right).$$

**Notation Convention**. When *r* is fixed throughout a discussion, we omit this argument and write *a*_α_, *b*_β_, *t*_αβ_, *h*_αβ_(*t*), *c*(*t*) in place of *a*_α*r*_, *b*_β*r*_, *t*_αβ*r*_, *h*_αβ*r*_(*t*), *c*_*r*_(*t*). (For binary factors α, β, we also conveniently replace α, β in indexation with *i*, *j*.)

Following this convention, there are three different arrangements of *a*_1_, *a*_2_, *b*_1_, *b*_2_ (for a given *R* = *r*) satisfying (11)–(12):

(14) $$\begin{array}{c} \;(i)\;a_{1}\leq b_{1}\leq a_{2}\leq b_{2}\\ (ii)\;a_{1}\leq a_{2}\leq b_{1}\leq b_{2}\\ (iii)\;a_{1}\leq b_{1}\leq b_{2}\leq a_{2} \end{array}$$

In all three cases,

(15) $$t_{11}=\min\left(a_{1},b_{1}\right)=a_{1}=\min\left(a_{1},b_{2}\right)=t_{12}.$$

For arrangement (i) we have

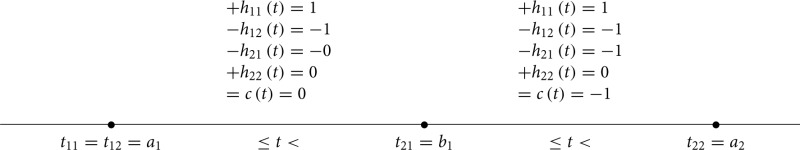



This diagram shows the values of *h*_*ijr*_(*t*) and the resulting values of *c*_*r*_(*t*) as *t* changes with respect to the fixed positions of *t*_*ijr*_ (with index *r* dropped everywhere). Analogously, for arrangements (ii) and (iii), we have, respectively

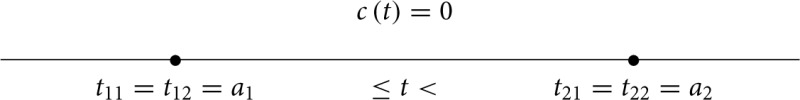



and

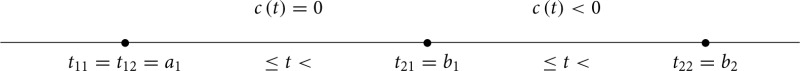



In all three cases, *c*(*t*) is obviously zero for *t* < *t*_11_ and *t* ≥ *t*_22_. We see that *c*(*t*) = *c*_*r*_(*t*) ≤ 0 for all *t* and every *R* = *r*. It follows that *C*(*t*) ≤ 0, because

(16) $$\mathrm{Pr}\left(T_{ij}\leq t\right)=\int h_{ijr}\left(t\right)\text{d}\mu \left(r\right),$$

for *i*, *j* ∈ {1, 2}, and

(17) $$C\left(t\right)=\int c_{r}\left(t\right)\text{d}\mu \left(r\right)\leq 0,$$

where μ is the probability measure associated with *R* and the integration is over all possible *r*. We obtain the same result as in (5), but in a very different way.

In this paper we extend this approach to other mental architectures belonging to the class of series-parallel networks, those involving other composition operations and possibly more than just two selectively influenced processes. In doing so we follow a long trail of work mentioned earlier. When dealing with multiple processes we follow Yang et al. (2013) in using high-order interaction contrasts. All our results are replications or straightforward generalizations of the results already known: the primary value of our work therefore is not in characterizing mental architectures, but rather in demonstrating a new theoretical approach and a new proof technique.

### 1.1. Definitions, terminology, and notation

Since we deal with the durations of processes rather than the processes themselves, we use the term *composition* to describe a function that relates the durations of the components of a network to the overall (observed) duration. Formally, a composition is a real-valued function *t* = *t*(*a*, *b*, …, *z*) of an arbitrary number of real-valued arguments. The arguments *a*, *b*, …, *z* are referred to as *durations* or *components*. In this article, we will use *X* ∧ *Y* ∧ … ∧ *Z* to denote min(*X*, *Y*, …, *Z*), and *X* ∨ *Y* ∨ … ∨ *Z* to denote max(*X*, *Y* …, *Z*).

A *series-parallel composition* (SP) is defined as follows.

**Definition 1.2**. (1) A single duration is an SP composition. (2) If *X* and *Y* are SP compositions with disjoint sets of arguments, then *X* ∧ *Y*, *X* ∨ *Y*, and *X* + *Y* are SP compositions. (3) There are no other SP compositions than those construable by Rules 1 and 2.

*Remark* 1.3. The requirement that *X* and *Y* in Rule 2 have disjoint sets of arguments prevents expressions like *X* ∧ *X* or *X* + *X* ∨ *Y*. But if the second *X* in *X* ∧ *X* is renamed into *X*′, or *X* ∨ *Y* in *X* + *X* ∨ *Y* is renamed into *Z*, then the resulting *X* ∧ *X*′ and *X* + *Z* are legitimate SP compositions. This follows from the generality of our treatment, in which different components of an SP composition may have arbitrary joint distributions: e.g., *X* and *X*′ in *X* ∧ *X*′ may very well be jointly distributed so that Pr[*X* = *X*′] = 1. One should, however, always keep in minds the pattern of selective influences: thus, if *X* is influenced by α, then *Z* is also influenced by α in *X* + *Z* above.

Any SP composition is obtained by a successive application of Rules 1 and 2 (the sequence being generally non-unique), and at any intermediate stage of such a sequence we also have an SP composition that we can term a *subcomposition*.

**Definition 1.4**. Two durations *X*, *Y* in an SP composition are said to be *parallel* or *concurrent* if there is a subcomposition of this SP composition of the form SP^1^(*X*, *X*′, …) ∧ SP^2^(*Y*, *Y*′, …) (in which case *X*, *Y* are said to be *min-parallel*) or SP^1^(*X*, *X*′, …) ∨ SP^2^(*Y*, *Y*′, …) (*X*, *Y* are *max-parallel*). *X*, *Y* in an SP composition are said to be *sequential* or *serial* if there is a subcomposition of this SP composition of the form SP^1^(*X*, *X*′, …) + SP^2^(*Y*, *Y*′, …).

**Definition 1.5**. An SP composition is called *homogeneous* if it does not contain both ∧ and ∨ in it; if it does not contain ∧, it is denoted SP_∨_; if it does not contain ∨, it is denoted SP_∧_.

The only SP composition that is both SP_∧_ and SP_∨_ is a purely serial one: *a* + *b* + … + *z*. Most of the results previously obtained for mental networks are confined to homogeneous compositions. We will not need this constraint for the most part.

Since we will be dealing with compositions of more than just two components, we need to extend the definition of selective influences mentioned above. In the formulation below, ~ stands for “has the same distribution as.” A treatment ϕ =(λ^1^_*i*_1__, …, λ^*n*^_*i*_*n*__) is a vector of values of the factors λ^1^, …, λ^*n*^, the values of λ^*k*^ (*k* = 1, …, *n*) being indicated by subscripts, λ^*k*^_*i*_*k*__.

**Definition 1.6**. Random variables (*X*^1^, …, *X*^*n*^) are *selectively influenced* by factors (λ^1^, …, λ^*n*^), respectively,

(18) $$(X^{1},\ldots ,X^{n})↫(\lambda^{1},\ldots ,\lambda^{n}),$$

if for some random variable *R*, whose distribution does not depend on (λ^1^, … λ^*n*^), and for some functions *g*_1_, …, *g*_*n*_,

(19) $$(X_{\phi }^{1},\ldots ,X_{\phi }^{n})\sim (g_{1}(\lambda_{i_{1}}^{1},R),\ldots ,g_{n}(\lambda_{i_{n}}^{n},R)),$$

for any treatment $\phi =\left(\lambda_{i_{1}}^{1},\ldots ,\lambda_{i_{n}}^{n}\right)$.

In the subsequent discussion we assume that all non-dummy factors involved are binary in a completely crossed design (i.e., the overall time *T* is recorded for all 2^*n*^ vectors of values for ϕ). When we have random variables not influenced by any of these factors, we will say they selectively influenced by an empty set of factors (we could also, equivalently, introduce for them dummy factors, with one value each).

## 2. SP compositions containing two selectively influenced processes

Consider two processes, with durations *A* and *B* in an SP composition. The overall duration of this SP composition can be written as a function of *A*, *B* and other components: *T* = *T*(*A*, *B*, …). We assume that *A*, *B*, and all other components are selectively influenced by α, β, and empty set, respectively: (*A*, *B*, …) ↫ (α, β, ∅). Let each factor has two levels: α = 1, 2 and β = 1, 2, with four allowable treatments (1, 1), (1, 2), (2, 1), and (2, 2). The corresponding overall durations (random variables) are written as *T*_11_, *T*_12_, *T*_21_, and *T*_22_.

By Definition 1.6 of selective influences, each process duration (a random variable) is a function of some random variable *R* and the corresponding factor: *A* = *a*(α, *R*), *B* = *b*(β, *R*). For any given value *R* = *r*, the component durations are fixed numbers,

(20) $$\begin{array}{l} a\left(\alpha =1,r\right)=a_{1r},\;a\left(\alpha =2,r\right)=a_{2r},\\ b\left(\beta =1,r\right)=b_{1r},\;b\left(\beta =2,r\right)=b_{2r},\\ \;x\left(\emptyset ,r\right)=x_{r}, \end{array}$$

where *x* is the value of any duration *X* in the composition other than *A* and *B*. We assume that *R* is chosen so that the Prolongation Assumption (11) holds, with the convention (12).

The overall duration *T* at *R* = *r* is also a fixed number, written as (recall that we replace α, β in indexation with *i*, *j*)

(21) $$T\left(a_{ir},\beta_{jr},\ldots \right)=t_{ijr},i,j\in \left\{1,2\right\}.$$

The distribution function for *t*_*ijr*_ is the shifted Heaviside function *h*_*ijr*_ (*t*) = *h*(*t* − *t*_*ijr*_),

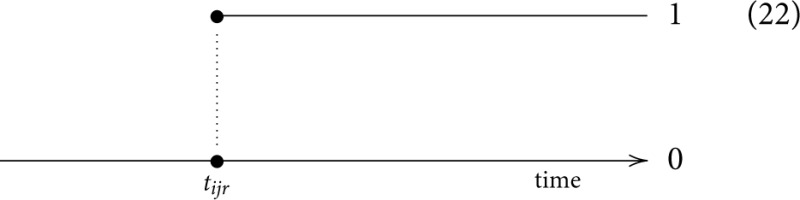



The *conditional interaction contrast c*_*r*_(*t*) is defined by (13). Denoting by *H*_*ij*_(*t*) the distribution function of *T*_*ij*_, we have

(23) $$H_{ij}\left(t\right)=\int_{ℛ}h_{ijr}\left(t\right)\text{d}\mu_{r},$$

with ℛ denoting the set of possible values of *R*. For the observable (i.e., estimable from data) interaction contrast

(24) $$C\left(t\right)=H_{11}\left(t\right)-H_{12}\left(t\right)-H_{21}\left(t\right)+H_{22}\left(t\right),$$

we have then

(25) $$C\left(t\right)=\int_{ℛ}c_{r}\left(t\right)\text{d}\mu_{r}.$$

Note that it follows from our Prolongation Assumption that

(26) $$\begin{array}{l} H_{11}\left(t\right)\geq H_{12}\left(t\right),\;H_{21}\left(t\right)\geq H_{22}\left(t\right),\\ H_{11}\left(t\right)\geq H_{21}\left(t\right),\;H_{12}\left(t\right)\geq H_{22}\left(t\right). \end{array}$$

We also define two conditional cumulative interaction contrasts (conditioned on *R* = *r*):

(27) $$c\left(0,t\right)=\int_{0}^{t}c\left(\tau \right)\text{d}\tau .$$

(28) $$c\left(t,\infty \right)=\int_{t}^{\infty }c\left(\tau \right)\text{d}\tau =\underset{u\to \infty }{\lim}\int_{t}^{u}c\left(\tau \right)\text{d}\tau .$$

The corresponding observable cumulative interaction contrasts are

(29) $$\begin{array}{l} C\left(0,t\right)=\int_{ℛ}c\left(0,t\right)\text{d}\mu_{r}=\int_{ℛ}\left(\int_{0}^{t}c\left(\tau \right)\text{d}\tau \right)\text{d}\mu_{r}\\ \;=\int_{0}^{t}\left(\int_{ℛ}c\left(\tau \right)\text{d}\mu_{r}\right)\text{d}\tau =\int_{0}^{t}C\left(\tau \right)\text{d}\tau . \end{array}$$

(30) $$\begin{array}{l} C\left(t,\infty \right)=\int_{ℛ}c\left(t,\infty \right)\text{d}\mu_{r}=\int_{ℛ}\left(\int_{t}^{\infty }c\left(\tau \right)\text{d}\tau \right)\text{d}\mu_{r}\\ \;=\int_{t}^{\infty }\left(\int_{ℛ}c\left(\tau \right)\text{d}\mu_{r}\right)\text{d}\tau =\int_{t}^{\infty }C\left(\tau \right)\text{d}\tau . \end{array}$$

In these formulas we could switch the order of integration by Fubini's theorem, because, for any interval of reals *I*,

(31) $$\int_{I\times ℛ}\left|c\left(\tau \right)\right|\text{d}\left(\tau \times \mu_{r}\right)\leq \int_{I\times ℛ}2\text{d}\left(\tau \times \mu_{r}\right)\leq 2.$$

### 2.1. Four Lemmas

Recall the definition of *c*_*r*_(*t*) in (13). We follow our Notation Convention and drop the index *r* in *c*_*r*_(*t*) and all other expressions for a fixed *r*.

**Lemma 2.1**. *In any* SP *architecture, for any r,*

$$t_{11}\leq t_{12}\wedge t_{21}\leq t_{12}\vee t_{21}\leq t_{22}.$$

*Proof*. Follows from the (nonstrict) monotonicity of the SP composition in all arguments.           □

**Lemma 2.2**. *In any* SP *architecture, for any r, c*(*t*) *equals 0 for all values of t except for two cases:*

$$({\text{Case}}^{+})if\;t_{11}\leq t<t_{12}\wedge t_{21},\;then\;c\left(t\right)=1-0-0+0>0,$$

and

$$({\text{Case}}^{-})if\;t_{12}\vee t_{21}\leq t<t_{22},then\;c\left(t\right)=1-1-1+0<0.$$

*Proof*. By direct computation.                       □

**Lemma 2.3**. *In any* SP *architecture, for any r, c* (*t*) ≤ 0 *for all values of t if and only if t*_11_ = *t*_12_ ∧ *t*_21_; *c*(*t*) ≥ 0 *for all values of t if and only if t*_12_ ∨ *t*_21_ = *t*_22_.

*Proof*. Immediately follows from Lemma 2.2.                       □

**Lemma 2.4**. *In any* SP *architecture, for any r*,

- $c\left(0,t\right)=\int_{0}^{t}c\left(\tau \right)d\tau \geq 0$ *for any t if and only if* −*t*_11_ + *t*_12_ + *t*_21_ − *t*_22_ ≥ 0,
- $c\left(t,\infty \right)=\int_{t}^{\infty }c\left(\tau \right)d\tau \leq \text{ ​​}0$ *for any t if and only if* −*t*_11_ + *t*_12_ + *t*_21_ − *t*_22_ ≤ 0,
- lim_*t*→∞_*c*(*0*, *t*) = 0 *if and only if* −*t*_11_+*t*_12_+*t*_21_−*t*_22_ = 0.
- lim_*t*→0_*c*(*t*, ∞) = 0 *if and only if* −*t*_11_+*t*_12_+*t*_21_−*t*_22_ = 0.

*Proof*. Without loss of generality, put *t*_12_ ≤ *t*_21_. We have

$$c\left(0,t\right)=\{\begin{array}{ll} 0 & \text{if }t<t_{11}\\ \left(t-t_{11}\right) & \text{if }t_{11}\leq t<t_{12}\\ \left(t-t_{11}\right)-\left(t-t_{12}\right)=t_{12}-t_{11} & \text{if }t_{12}\leq t<t_{21}\\ \left(t-t_{11}\right)-\left(t-t_{12}\right)-\left(t-t_{21}\right) & \\ \;=-t_{11}+t_{12}+t_{21}-t & \text{if }t_{21}\leq t<t_{22}\\ \left(t-t_{11}\right)-\left(t-t_{12}\right)-\left(t-t_{21}\right)+\left(t-t_{22}\right) & \\ \;=-t_{11}+t_{12}+t_{21}-t_{22} & \text{if }t\geq t_{22} \end{array}$$

The expressions for the first three cases are obviously nonnegative. If −*t*_11_ + *t*_12_ + *t*_21_ − *t*_22_ ≥ 0, then *c*(0, *t*) ≥ 0 for all *t* in the last case (*t* ≥ *t*_22_). With −*t*_11_ + *t*_12_ + *t*_21_ − *t*_22_ ≥ 0, we have −*t*_11_ + *t*_12_ + *t*_21_ − *t* ≥ *t*_22_ − *t* ≥ 0 for the fourth case (*t*_21_ ≤ *t* < *t*_22_). Hence *c*(0, *t*) ≥ 0 for all *t* if −*t*_11_ + *t*_12_ + *t*_21_ − *t*_22_ ≥ 0. Conversely, if *c*(0, *t*) ≥ 0 for all *t*, then it is also true for *t* ≥ *t*_22_, whence −*t*_11_ + *t*_12_ + *t*_21_ − *t*_22_ ≥ 0.

The proof for $c\left(t,\infty \right)=\int_{t}^{\infty }c\left(\tau \right)d\tau$ requires replacing it first with $\int_{t}^{u}c\left(\tau \right)d\tau \leq 0$ for some *u* > *t*_22_. We have

$$\int_{t}^{u}c\left(\tau \right)d\tau =\{\begin{array}{ll} \left(u-t_{11}\right)-\left(u-t_{12}\right)-\left(u-t_{21}\right)+\left(u-t_{22}\right) & \\ \;=-t_{11}+t_{12}+t_{21}-t_{22} & \text{if }t<t_{11}\\ \left(u-t\right)-\left(u-t_{12}\right)-\left(u-t_{21}\right)+\left(u-t_{22}\right) & \\ \;=-t+t_{12}+t_{21}-t_{22} & \text{if }t_{11}\leq t<t_{12}\\ \left(u-t\right)-\left(u-t\right)-\left(u-t_{21}\right)+\left(u-t_{22}\right) & \\ \;=t_{21}-t_{22} & \text{if }t_{12}\leq t<t_{21}\\ \left(u-t\right)-\left(u-t\right)-\left(u-t\right)+\left(u-t_{22}\right) & \\ \;=t-t_{22} & \text{if }t_{21}\leq t<t_{22}\\ \left(u-t\right)-\left(u-t\right)-\left(u-t\right)+\left(u-t\right) & \\ \;=0 & \text{if }t\geq t_{22} \end{array}$$

The expressions for the last three cases are obviously nonpositive. If −*t*_11_ + *t*_12_ + *t*_21_ − *t*_22_ ≤ 0, then $\int_{t}^{u}c^{\left(2\right)}\left(\tau \right)d\tau \leq 0$ for all *t* in the first case (*t* < *t*_11_). With −*t*_11_ + *t*_12_ + *t*_21_ − *t*_22_ ≤ 0, we have −*t* + *t*_12_ + *t*_21_ − *t*_22_ ≤ *t*_11_ − *t* < 0 for the second case (*t*_11_ ≤ *t* < *t*_12_). Hence $\int_{t}^{u}c\left(\tau \right)d\tau \leq 0$ for all *t* if −*t*_11_ + *t*_12_ + *t*_21_ − *t*_22_ ≤ 0. Since in all expressions *u* is algebraically eliminated, they remain unchanged as *u* → ∞. Conversely, if *c*(*t*, ∞) ≤ 0 for all *t*, then it is also true for *t* < *t*_11_, whence −*t*_11_ + *t*_12_ + *t*_21_ − *t*_22_ ≤ 0.

The statements (iii) and (iv) follow trivially.                       □

### 2.2. Parallel processes

#### 2.2.1. Simple parallel architectures of size 2

A simple parallel architecture corresponds to one of the two compositions: *T* = *A* ∧ *B* or *T* = *A* ∨ *B*, with (*A*, *B*) ↫ (α, β). Recall the definition of *C*(*t*) in (24).

**Theorem 2.5**. *For T* = *A* ∧ *B*, *we have c*(*t*) ≤ 0 *for any r*, *t*; *consequently*, *C*(*t*) ≤ 0 *for any t*. *For T* = *A* ∨ *B*, *we have c*(*t*) ≥ 0 *for any r*, *t*; *consequently, C*(*t*) ≥ 0 *for any t*.

*Proof*. For *T* = *A* ∧ *B* with the Prolongation Assumption (11)–(12), we have

$$t_{11}=a_{1}\wedge b_{1}=a_{1},\;t_{12}=a_{1}\wedge b_{2},\;t_{21}=a_{2}\wedge b_{1}.$$

It follows that

$$t_{12}\wedge t_{21}=a_{1}\wedge b_{2}\wedge a_{2}\wedge b_{1}=a_{1}=t_{11}.$$

By Lemma 2.3, *c*(*t*) ≤ 0. As *C*(*t*) in (25) preserves the sign of *c*(*t*), we have *C*(*t*) ≤ 0. For *T* = *A* ∧ *B*, we have

$$t_{22}=a_{2}\vee b_{2},\;t_{12}=a_{1}\vee b_{2},\;t_{21}=a_{2}\vee b_{1}.$$

It follows that

$$t_{12}\vee t_{21}=a_{1}\vee b_{2}\vee a_{2}\vee b_{1}=t_{22},$$

whence, by Lemma 2.3, *c*(*t*) ≥ 0 and therefore *C*(*t*) ≥ 0.                       □

#### 2.2.2. Two parallel processes in an arbitrary SP network

Consider now a composition SP(*A*, *B*, …) with (*A*, *B*, …) ↫ (α, β, ∅).

**Lemma 2.6**. *If A*, *B in* SP(*A*, *B*, …) *are parallel, then* SP(*A*, *B*, …) *can be presented as A*′ ∧ *B*′ *if they are min-parallel, or as A*′ ∨ *B*′ *if they are max-parallel, so that* (*A*′, *B*′) ↫ (α, β) *and, for any fixed R* = *r*, *the Prolongation Assumption holds*.

*Proof*. By Definitions 1.2 and 1.4, if *A*, *B* are min-parallel, then SP_∧_(*A*, *B*, …) can be presented either as

$${\text{SP}}^{1}(A,\ldots )\wedge {\text{SP}}^{2}(B,\ldots )$$

or

$$\left({\text{SP}}^{1}(A,\ldots )\wedge {\text{SP}}^{2}(B,\ldots )+X\right)\wedge Y,$$

or else

$$\left({\text{SP}}^{1}(A,\ldots )\wedge {\text{SP}}^{2}(B,\ldots )\wedge X\right)+Y,$$

where *B* does not enter in SP^1^ and *A* does not enter in SP^2^. On renaming

$$\underset{=A^{\prime }}{\underset{︸}{{\text{SP}}^{1}(A,\ldots )}}\wedge \underset{=B^{\prime }}{\underset{︸}{{\text{SP}}^{2}(B,\ldots )}},$$

$$\begin{array}{l} \left({\text{SP}}^{1}(A,\ldots )\wedge {\text{SP}}^{2}(B,\ldots )+X\right)\wedge Y\\ \;=\underset{=A^{\prime }}{\underset{︸}{\left({\text{SP}}^{1}(A,\ldots )+X\right)}}\wedge \underset{=B^{\prime }}{\underset{︸}{\left({\text{SP}}^{2}(B,\ldots )+X\right)\wedge Y}}, \end{array}$$

and

$$\begin{array}{l} \left({\text{SP}}^{1}(A,\ldots )\wedge {\text{SP}}^{2}(B,\ldots )\wedge X\right)+Y\\ \;=\underset{=A^{\prime }}{\underset{︸}{\left({\text{SP}}^{1}(A,\ldots )+Y\right)}}\wedge \underset{=B^{\prime }}{\underset{︸}{\left({\text{SP}}^{2}(B,\ldots )\wedge X+Y\right)}}, \end{array}$$

we have, obviously, (*A*′, *B*′) ↫ (α, β). Fixing *R* = *r*, by the (nonstrict) monotonicity of SP compositions,

$${a^{\prime }}_{1}={\text{SP}}^{1}(a_{1},\ldots )\leq {\text{SP}}^{1}(a_{2},\ldots )={a^{\prime }}_{2}$$

and

$${b^{\prime }}_{1}={\text{SP}}^{2}(b_{1},\ldots )\leq {\text{SP}}^{2}(b_{2},\ldots )={b^{\prime }}_{2}$$

We can also put *a*′_1_ = SP^1^(*a*_1_, …) ≤ SP^2^(*b*_1_, …) = *b*′_1_ (otherwise we can rename the variables). The proof for the max-parallel case is analogous.

**Theorem 2.7**. *If A*, *B in* SP(*A*, *B*, …) *are min-parallel, then c*(*t*) ≤ 0 *for any r*, *t*; *consequently, C*(*t*) ≤ 0 *for any t. If A*, *B in* SP(*A*, *B*, …) *are max-parallel, then c*(*t*) ≥ 0 *for any r*, *t*; *consequently, C*(*t*) ≥ 0 *for any t*.

*Proof*. Immediately follows from Lemma 2.6 and Theorem 2.5.                       □

### 2.3. Sequential processes

#### 2.3.1. Simple serial architectures of size 2

Simple serial architectures of size 2 corresponds to the SP composition *T* = *A* + *B*, with (*A*, *B*) ↫ (α, β). Recall the definitions of the two cumulative interaction contrasts: (27)–(30).

**Theorem 2.8**. *If T* = *A* + *B*, *then c*(0, *t*) ≥ 0 *and c*(*t*, ∞) ≤ 0 *for any r*, *t*; *moreover*,

$$\underset{t\to \infty }{\lim}c\left(0,t\right)=\underset{t\to 0}{\lim}c\left(t,\infty \right)=0,$$

*for any r*, *t*. *Consequently, C*(0, *t*) ≥ 0, *C*(*t*, ∞) ≤ 0 *for any t, and*

$$\underset{t\to \infty }{\lim}C\left(0,t\right)=\underset{t\to 0}{\lim}C\left(t,\infty \right)=0$$

*Proof*. Follows immediately from Lemma 2.4, since

$$\begin{array}{l} -t_{11}+t_{12}+t_{21}-t_{22}=-\left(a_{1}+b_{1}\right)+\left(a_{1}+b_{2}\right)\\ \;+\left(a_{2}+b_{1}\right)-\left(a_{2}+b_{2}\right)=0. \end{array}$$

                               □

#### 2.3.2. Two sequential processes in an arbitrary SP network

Consider now a composition SP(*A*, *B*, …) with (*A*, *B*, …) ↫ (α, β, ∅).

**Theorem 2.9**. *If A and B are sequential in an* SP(*A*, *B*, …) *composition, then one or both of the following statements hold*:

- *c*(0, *t*) ≥ 0 *for any r*, *t*, *and C*(0, *t*) ≥ 0 *for any t*,
- *c*(*t*, ∞) ≤ 0 *for any r*, *t*, *and C*(*t*, ∞) ≤ 0 *for any t*.

*Proof*. In accordance with Definitions 1.2 and 1.4, SP(*A*, *B*, …) with sequential *A*, *B* can be presented as either

(32) $$\left({\text{SP}}^{1}(A,\ldots )+{\text{SP}}^{2}(B,\ldots )\right)\wedge X+Y$$

or

(33) $$\left({\text{SP}}^{1}(A,\ldots )+{\text{SP}}^{2}(B,\ldots )\right)\vee X+Y$$

(note that any *Z* in SP^1^(*A*, …) + SP^2^(*B*, …) + *Z* can be absorbed by either of the first two summands). For both cases, by the monotonicity of SP compositions, for any *R* = *r*, SP^1^(*a*_1_, …) ≤ SP^1^(*a*_2_, …), SP^2^(*b*_1_, …) ≤ SP^2^(*b*_2_, …), and we can always assume SP^1^(*a*_1_, …) ≤ SP^2^(*b*_1_, …). Denoting the durations of SP^1^(a_i_, …) + SP^2^(*b*_*j*_, …) by *t*′_*ij*_, we have therefore, by Theorem 2.8, −*t*′_11_ + *t*′_12_ + *t*′_21_ − *t*′_22_ = 0. Denoting the durations of *X* and *Y* by *t*′ and *t*″, respectively, in the case (32) we have

$$t_{ij}={t^{\prime }}_{ij}\wedge t^{\prime }+t^{\prime \prime }.$$

By Lemma 2.4, all we have to show is that −*t*_11_+*t*_12_+*t*_21_−*t*_22_ ≥ 0. It is easy to see that *t*″ does not affect this linear combination, and its value is (assuming *t*′_12_ ≤ *t*′_21_, without loss of generality)

$$\{\begin{array}{ll} 0 & \text{if }t^{\prime }<{t^{\prime }}_{11}\\ -{t^{\prime }}_{11}+t^{\prime } & \text{if }{t^{\prime }}_{11}\leq t^{\prime }<{t^{\prime }}_{12}\\ -{t^{\prime }}_{11}+{t^{\prime }}_{12} & \text{if }{t^{\prime }}_{12}\leq t^{\prime }<{t^{\prime }}_{21}\\ -{t^{\prime }}_{11}+{t^{\prime }}_{12}+{t^{\prime }}_{21}-t^{\prime } & \text{if }{t^{\prime }}_{21}\leq t^{\prime }<{t^{\prime }}_{22}\\ -{t^{\prime }}_{11}+{t^{\prime }}_{12}+{t^{\prime }}_{21}-{t^{\prime }}_{22} & \text{if }t^{\prime }\geq {t^{\prime }}_{22}. \end{array}$$

The nonnegativity of the first three expressions is obvious, the fifth one is zero, and the forth expression is larger than the fifth because *t*′ < *t*′_22_.

The proof for the case (33) is analogous.                       □

If the SP composition with sequential *A*, *B* is homogeneous (Definition 1.5), the statement of theorem can be made more specific.

**Theorem 2.10**. *If A and B are sequential in an* SP_∧_(*A*, *B*, …) *composition, then c*(0, *t*) ≥ 0 *for any r*, *t*, *and C*(0, *t*) ≥ 0 *for any t*; *if the composition is* SP_∨_(*A*, *B*, …), *then c*(*t*, ∞) ≤ 0 *for any r*, *t*, *and C*(*t*, ∞) ≤ 0 *for any t*.

## 3. Multiple processes

We now turn to networks containing *n* ≥ 2 processes with durations (*X*^1^, …, *X*^*n*^), selectively influenced by factors (λ^1^, …, λ^*n*^). In other words, we deal with compositions SP(*X*^1^, …, *X*^*n*^, …) such that (*X*^1^, …, *X*^*n*^, …) ↫ (λ^1^, …, λ^*n*^, ∅), where each λ^*k*^ is binary, with values 1,2. There are 2^*n*^ allowable treatments and 2^*n*^ corresponding overall durations, *T*_11…1_, *T*_11…2_, …, *T*_22…2_. According to Definition 1.6 of selective influences, each process duration here is a function of some random variable *R* and of the corresponding factor, *X*^*k*^ = *x*^*k*^(*R*, λ^*k*^). For any fixed value *R* = *r*, these durations are fixed numbers for any given treatment, and so is the overall, observed value of the SP composition. We denote them

(34) $$x^{k}(r,\lambda^{k}=1)=x_{1r}^{k},\;x^{k}(r,\lambda^{k}=2)=x_{2r}^{k},$$

and

(35) $$T(x_{i_{1}r}^{1},x_{i_{2}r}^{2},\ldots ,x_{i_{n}r}^{n},\ldots ),\ldots =t_{i_{1}i_{2}...i_{n}r},$$

where *i*_1_, *i*_2_, …, *i*_*n*_ ∈ {1, 2}. The distribution function for *t*_*i*_1_*i*_2_…*i*_*n*_*r*_ is a shifted Heaviside function

(36) $$h_{i_{1}i_{2}...i_{n}r}\left(t\right)=\{\begin{array}{c} 0,\;if\;t<t_{i_{1}i_{2}...i_{n}r}\\ 1,\;if\;t\geq t_{i_{1}i_{2}...i_{n}r} \end{array}.$$

Denoting by *H*_*i*_1_*i*_2_…*i*_*n*__(*t*) the distribution function of *T*_*i*_1_*i*_2_…*i*_n__, we have

(37) $$H_{i_{1}i_{2}...i_{n}}\left(t\right)=\int_{ℛ}h_{i_{1}i_{2}...i_{n}r}\left(t\right)\text{d}\mu_{r}.$$

Conditioned on *R* = *r*, the *n*-*th order interaction contrast* is defined in terms of mixed finite differences as

(38) $$c_{r}^{\left(n\right)}\left(t\right)=\Delta_{i_{1}}\Delta_{i_{2}}\ldots \Delta_{i_{n}}h_{i_{1}i_{2}...i_{n}r}\left(t\right),$$

which, with some algebra can be shown to be equal to

(39) $$c_{r}^{\left(n\right)}\left(t\right)=\sum_{i_{1},i_{2},\ldots ,i_{n}}{\left(-1\right)}^{n+\sum_{k=1}^{n}i_{k}}h_{i_{1}\ldots i_{n}r}\left(t\right).$$

Thus,

(40) $$\begin{array}{l} c_{r}^{\left(1\right)}\left(t\right)=\Delta_{i_{1}}h_{i_{1}r}\left(t\right)=h_{1r}\left(t\right)-h_{2r}\left(t\right)\\ \;=\sum_{i_{1}}{\left(-1\right)}^{1+i_{1}}h_{i_{1}r}\left(t\right), \end{array}$$

(41) $$\begin{array}{l} c_{r}^{\left(2\right)}\left(t\right)=\Delta_{i_{1}}\Delta_{i_{2}}h_{i_{1}i_{2}r}\left(t\right)=\left[h_{11r}\left(t\right)-h_{12r}\left(t\right)\right]\\ \;-\left[h_{21r}\left(t\right)-h_{22r}\left(t\right)\right]\\ \;=h_{11r}\left(t\right)-h_{12r}\left(t\right)-h_{21r}\left(t\right)+h_{22r}\left(t\right)\\ \;=\sum_{i_{1},i_{2}}{\left(-1\right)}^{2+i_{1}+i_{2}}h_{i_{1}i_{2}r}\left(t\right), \end{array}$$

(42) $$\begin{array}{l} \;c_{r}^{\left(3\right)}\left(t\right)=\Delta_{i_{1}}\Delta_{i_{2}}\Delta_{i_{3}}h_{i_{1}i_{2}i_{3}r}\left(t\right)\\ =\left\{\left[h_{111r}\left(t\right)-h_{112r}\left(t\right)\right]-\left[h_{121r}\left(t\right)-h_{122r}\left(t\right)\right]\right\}\\ -\left\{\left[h_{211r}\left(t\right)-h_{212r}\left(t\right)\right]-\left[h_{221r}\left(t\right)-h_{222r}\left(t\right)\right]\right\}\\ \;=h_{111r}\left(t\right)-h_{112r}\left(t\right)-h_{121r}\left(t\right)-h_{211r}\left(t\right)\\ \;+h_{122r}\left(t\right)+h_{212r}\left(t\right)+h_{221r}\left(t\right)-h_{222r}\left(t\right)\\ \;=\sum_{i_{1},i_{2},i_{3}}{\left(-1\right)}^{3+i_{1}+i_{2}+i_{3}}h_{i_{1}i_{2}i_{3}r}\left(t\right), \end{array}$$

etc. The observable distribution function interaction contrast of order *n* is defined as

(43) $$C^{\left(n\right)}\left(t\right)=\int_{ℛ}c_{r}^{\left(n\right)}\left(t\right)\text{d}\mu_{r}.$$

By straightforward calculus this can be written in extenso as

(44) $$C^{\left(n\right)}\left(t\right)=\sum_{i_{1},i_{2},\ldots ,i_{n}}{\left(-1\right)}^{n+\sum_{k=1}^{n}i_{k}}H_{i_{1}\ldots i_{n}}\left(t\right),$$

or, in terms of finite differences,

(45) $$C^{\left(n\right)}\left(t\right)=\Delta_{i_{1}}\Delta_{i_{2}}\ldots \Delta_{i_{n}}H_{i_{1}i_{2}...i_{n}}\left(t\right).$$

This is essentially the high-order interaction contrast used by Yang et al. (2013), the only difference being that they use survivor functions 1 − *H*(*t*) rather than the distribution functions *H*(*t*). We see that *c*_*r*_(*t*) and *C*(*t*) in the preceding analysis correspond to *c*^(2)^_*r*_(*t*) and *C*^(2)^(*t*), respectively.

We also introduce *n*-*th order cumulative contrasts*. Conditioned on *R* = *r*, we define

(46) $$c_{r}^{\left[1\right]}\left(0,t\right)=c_{r}^{\left[1\right]}\left(t,\infty \right)=h_{1r}\left(t\right)-h_{2r}\left(t\right),$$

(47) $$c_{r}^{\left[2\right]}\left(0,t\right)=\int_{0}^{t}c_{r}^{\left(2\right)}\left(t_{1}\right)dt_{1},\;c_{r}^{\left[2\right]}\left(t,\infty \right)=\int_{t}^{\infty }c_{r}^{\left(2\right)}\left(t_{1}\right)dt_{1},$$

(48) $$\begin{array}{l} \;c_{r}^{\left[3\right]}\left(0,t\right)=\int_{0}^{t}\int_{0}^{t_{1}}c_{r}^{\left(3\right)}\left(t_{2}\right)dt_{2}dt_{1},\\ c_{r}^{\left[3\right]}\left(t,\infty \right)=\int_{t}^{\infty }\int_{t_{1}}^{\infty }c_{r}^{\left(3\right)}\left(t_{2}\right)dt_{2}dt_{1}, \end{array}$$

etc. Generalizing,

(49) $$c_{r}^{\left[n\right]}\left(0,t\right)=\int_{0}^{t}\left(\int_{0}^{t_{1}}\ldots \int_{0}^{t_{n-2}}c_{r}^{\left(n\right)}\left(t_{n-1}\right)dt_{n-1}\ldots dt_{2}\right)dt_{1},$$

(50) $$c_{r}^{\left[n\right]}\left(t,\infty \right)=\int_{t}^{\infty }\left(\int_{t_{1}}^{\infty }\ldots \int_{t_{n-2}}^{\infty }c_{r}^{\left(n\right)}\left(t_{n-1}\right)dt_{n-1}\ldots dt_{2}\right)dt_{1}.$$

The corresponding unconditional cumulative contrasts of the *n*-th order are, as always, defined by integration of the conditional ones:

(51) $$\begin{array}{l} \;C^{\left[n\right]}\left(0,t\right)=\int_{ℛ}c_{r}^{\left[n\right]}\left(0,t\right)\text{d}\mu_{r}\\ =\int_{0}^{t}\left(\int_{0}^{t_{1}}\ldots \int_{0}^{t_{n-2}}C^{\left(n\right)}\left(t_{n-1}\right)dt_{n-1}\ldots dt_{2}\right)dt_{1}, \end{array}$$

(52) $$\begin{array}{l} \;C^{\left[n\right]}\left(t,\infty \right)=\int_{ℛ}c_{r}^{\left[n\right]}\left(t,\infty \right)\text{d}\mu_{r}\\ =\int_{t}^{\infty }\left(\int_{t_{1}}^{\infty }\ldots \int_{t_{n-2}}^{\infty }C^{\left(n\right)}\left(t_{n-1}\right)dt_{n-1}\ldots dt_{2}\right)dt_{1}. \end{array}$$

In the proofs below we will make use of the recursive representation of the conditional cumulative contrasts *c*^[*n*]^_r_. It is verified by straightforward calculus. Denoting

(53) $$\begin{array}{l} c_{i_{w}r}^{\left(n-1\right)}\left(t\right)=\\ {\sum_{i_{1},...,i_{w-1},i_{w+1},...,i_{n}}\left(-1\right)}^{n-1-i_{w}+\sum_{k=1}^{n}i_{k}}h_{i_{1}...i_{w-1}i_{w}i_{w+1}...i_{n}r}\left(t\right), \end{array}$$

where *w* ∈ {1, …, *n*} and *i*_*w*_ is fixed at 1 or 2, we have:

(54) $$c_{r}^{\left[1\right]}\left(0,t\right)=c_{r}^{\left[1\right]}\left(t,\infty \right)=h_{1r}\left(t\right)-h_{2r}\left(t\right),$$

(55) $$\begin{array}{l} c_{r}^{\left[2\right]}\left(0,t\right)=\int_{0}^{t}c_{r}^{\left(2\right)}\left(\tau \right)d\tau \\ \;=\int_{0}^{t}\left(h_{11r}\left(\tau \right)-h_{12r}\left(\tau \right)-h_{21r}\left(\tau \right)+h_{22r}\left(\tau \right)\right)d\tau \\ \;=\int_{0}^{t}\left[c_{i_{w}=1,r}^{\left(1\right)}\left(\tau \right)-c_{i_{w}=2,r}^{\left(1\right)}\left(\tau \right)\right]d\tau \\ \;=\int_{0}^{t}c_{i_{w}=1,r}^{\left[1\right]}\left(0,\tau \right)d\tau -\int_{0}^{t}c_{i_{w}=2,r}^{\left[1\right]}\left(0,\tau \right)d\tau , \end{array}$$

(56) $$\begin{array}{l} c_{r}^{\left[2\right]}\left(t,\infty \right)=\int_{t}^{\infty }c_{r}^{\left(2\right)}\left(\tau \right)d\tau \\ \;=\int_{t}^{\infty }\left(h_{11r}\left(\tau \right)-h_{12r}\left(\tau \right)-h_{21r}\left(\tau \right)+h_{22r}\left(\tau \right)\right)d\tau \\ \;=\int_{t}^{\infty }\left[c_{i_{w}=1,r}^{\left(1\right)}\left(\tau \right)-c_{i_{w}=2,r}^{\left(1\right)}\left(\tau \right)\right]d\tau \\ \;=\int_{t}^{\infty }c_{i_{w}=1,r}^{\left[1\right]}\left(\tau ,\infty \right)d\tau -\int_{t}^{\infty }c_{i_{w}=2,r}^{\left[1\right]}\left(\tau ,\infty \right)d\tau , \end{array}$$

(57) $$\begin{array}{l} c_{r}^{\left[3\right]}\left(0,t\right)=\int_{0}^{t}\int_{0}^{t_{1}}c_{r}^{\left(3\right)}\left(t_{2}\right)dt_{2}dt_{1}\\ \;=\int_{0}^{t}\int_{0}^{t_{1}}\left[c_{i_{w}=1,r}^{\left(2\right)}\left(t_{2}\right)-c_{i_{w}=2,r}^{\left(2\right)}\left(t_{2}\right)\right]dt_{2}dt_{1}\\ \;=\int_{0}^{t}\left[\int_{0}^{t_{1}}c_{i_{w}=1,r}^{\left(2\right)}\left(t_{2}\right)dt_{2}-\int_{0}^{t_{1}}c_{i_{w}=2,r}^{\left(2\right)}\left(t_{2}\right)dt_{2}\right]dt_{1}\\ \;=\int_{0}^{t}c_{i_{w}=1,r}^{\left[2\right]}\left(0,\tau \right)d\tau -\int_{0}^{t}c_{i_{w}=2,r}^{\left[2\right]}\left(0,\tau \right)d\tau , \end{array}$$

(58) $$\begin{array}{l} c_{r}^{\left[3\right]}\left(t,\infty \right)=\int_{t}^{\infty }\int_{t_{1}}^{\infty }c_{r}^{\left(3\right)}\left(t_{2}\right)dt_{2}dt_{1}\\ \;=\int_{t}^{\infty }\int_{t_{1}}^{\infty }\left[c_{i_{w}=1,r}^{\left(2\right)}\left(t_{2}\right)-c_{i_{w}=2,r}^{\left(2\right)}\left(t_{2}\right)\right]dt_{2}dt_{1}\\ \;=\int_{t}^{\infty }\left[\int_{t_{1}}^{\infty }c_{i_{w}=1,r}^{\left(2\right)}\left(t_{2}\right)dt_{2}-\int_{t_{1}}^{\infty }c_{i_{w}=2,r}^{\left(2\right)}\left(t_{2}\right)dt_{2}\right]dt_{1}\\ \;=\int_{t}^{\infty }c_{i_{w}=1,r}^{\left[2\right]}\left(\tau ,\infty \right)d\tau -\int_{t}^{\infty }c_{i_{w}=2,r}^{\left[2\right]}\left(\tau ,\infty \right)d\tau , \end{array}$$

and generally, for *n* > 1,

(59) $$c_{r}^{\left[n\right]}\left(0,t\right)=\int_{0}^{t}c_{i_{w}=1,r}^{\left[n-1\right]}\left(0,\tau \right)d\tau -\int_{0}^{t}c_{i_{w}=2,r}^{\left[n-1\right]}\left(0,\tau \right)d\tau ,$$

(60) $$c^{\left[n\right]}\left(t,\infty \right)=\int_{t}^{\infty }c_{i_{w}=1,r}^{\left[n-1\right]}\left(\tau ,\infty \right)d\tau -\int_{t}^{\infty }c_{i_{w}=2,r}^{\left[n-1\right]}\left(\tau ,\infty \right)d\tau .$$

Also we have, by substitution of variables under integral,

(61) $$c_{i_{w}r}^{\left[n-1\right]}\left(0,t\right)=c_{r}^{\left[n-1\right]}\left(0,t-x_{i_{w}r}^{w}\right),$$

(62) $$c_{i_{wr}}^{\left[n-1\right]}\left(t,\infty \right)=c^{\left[n-1\right]}\left(t-x_{i_{w}r}^{w},\infty \right).$$

The Prolongation Assumption generalizing (11)–(12) is formulated as follows.

**Prolongation Assumption**. *R* and functions *x*^1^, …, *x*^*n*^ in (34) can be chosen so that *x*^*k*^_1*r*_ ≤ *x*^*k*^_2*r*_ for all *R* = *r* and for all *k* = 1, …, *n*. Without loss of generality, we can also assume *x*^1^_1*r*_ ≤ *x*^2^_1*r*_ ≤ … ≤ *x*^*n*^_1*r*_ (if not, rearrange *x*^1^_1*r*_, …, *x*^*n*^_1*r*_).

**Notation Convention**. As we did before for *n* = 2, when *r* is fixed throughout a discussion, we omit this argument and write *x*^1^_*i*_1__, …, *x*^*n*^_*i*_*n*__, *t*_*i*_1_**i**_2_…*i*_*n*__, *h*_*i*_1_*i*_2_…*i*_*n*__(*t*), *c*^(*n*)^(*t*) in place of *x*^1^_*i*_1_*r*_, …, *x*^*n*^_*i*_*n*_*r*_, *t*_*i*_1_*i*_2_…*i*_*n*_*r*_, *h*_*i*_1_*i*_2_…*i*_*n*_*r*_(*t*), *c*^(*n*)^_*r*_(*t*).

### 3.1. Parallel processes

#### 3.1.1. Simple parallel architectures of size *n*

**Theorem 3.1**. *If T* = *X*^1^ ∧ … ∧ *X*^*n*^, *then for any r*, *t*, *c*^(*n*)^(*t*) ≤ 0 *if n is even and c*^(*n*)^(*t*) ≥ 0 *if n is odd. Consequently, for any t*, *C*^(*n*)^(*t*) ≤ 0 *if n is even and C*^(*n*)^(*t*) ≥ 0 *if n is odd*.

*Proof*. By induction on *n*, the case *n* = 1 being true by the Prolongation Assumption:

$$c^{\left(1\right)}\left(t\right)=h_{1}\left(t\right)-h_{2}\left(t\right)\geq 0.$$

Let the statement of the theorem be true for *c*^(*n*−1)^(*t*), with *n* − 1 ≥ 1. By the Prolongation Assumption,

$$t_{1i_{2}...i_{n}}=x_{1}^{1}\wedge x_{i_{2}}^{2}\wedge \ldots \wedge x_{i_{n}}^{n}=x_{1}^{1},$$

for any *i*_2_ … *i*_*n*_, whence

$$h_{1i_{2}...i_{n}}\left(t\right)=\{\begin{array}{c} 0,\;if\;t<x_{1}^{1}\\ 1,\;if\;t\geq x_{1}^{1} \end{array}.$$

Therefore *c*^(*n*−1)^_*i*_1_=1_(*t*) = 0, and, applying the induction hypothesis to *c*^(*n*−1)^_*i*_1_=2_(*t*),

$$\begin{array}{l} c^{\left(n\right)}\left(t\right)=c_{i_{1}=1}^{\left(n-1\right)}\left(t\right)-c_{i_{1}=2}^{\left(n-1\right)}\left(t\right)=-c_{i_{1}=2}^{\left(n-1\right)}\left(t\right)\\ \;=\{\begin{array}{c} \leq 0,\text{ if }n\text{ is even}\\ \geq 0,\text{ if }n\text{ is odd} \end{array}. \end{array}$$

That *C*^(*n*)^(*t*) ≤ 0 if *n* is even and *C*^(*n*)^(*t*) ≥ 0 if *n* is odd follows by the standard argument.                       □

**Theorem 3.2**. *If T* = *X*^1^ ∨ … ∨ *X*^*n*^, *then for any r*, *t*, *c*^(*n*)^(*t*) ≥ 0. *Consequently, for any t*, *C*^(*n*)^(*t*) ≥ 0.

*Proof*. By induction on *n*, the case *n* = 1 being true by the Prolongation Assumption:

$$c^{\left(1\right)}\left(t\right)=h_{1}\left(t\right)-h_{2}\left(t\right)\geq 0.$$

Let the theorem be true for *c*^(*n*−1)^(*t*), where *n* − 1 ≥ 1. Let

$$x_{2}^{1}\vee x_{2}^{2}\vee \ldots \vee x_{2}^{n}=x_{2}^{m},$$

where 1 ≤ *m* ≤ *n*. We have then

$$t_{i_{1}i_{2}...i_{m-1}2i_{m+1}...i_{n}}=x_{2}^{m},$$

and

$$h_{i_{1}...i_{m-1}2i_{m+1}...i_{n}}(t)=\{\begin{array}{c} 0,\;if\;t<x_{2}^{m}\\ 1,\;if\;t\geq x_{2}^{m} \end{array},$$

for all *i*_1_…*i*_*m*−1_, *i*_*m* + 1_ … *i*_*n*_. Then *c*^(*n*−1)^_*i*_*m*_=2_(*t*) = 0, and

$$c^{\left(n\right)}\left(t\right)=c_{i_{m}=1}^{\left(n-1\right)}\left(t\right)-c_{i_{m}=2}^{\left(n-1\right)}\left(t\right)=c_{i_{m}=1}^{\left(n-1\right)}\left(t\right)\geq 0.$$

Consequently, *C*^(*n*)^(*t*) ≥ 0, for any *t*.                       □

#### 3.1.2. Multiple parallel processes in arbitrary SP networks

In a composition SP(*X*^1^, …, *X*^*n*^, …), the components *X*^1^, …, *X*^*n*^ are considered parallel if any two of them are parallel. We assume selective influences (*X*^1^, …, *X*^*n*^, …) ↫ (λ^1^, …, λ^*n*^, ∅). We do not consider the complex situation when some of the selectively influenced processes *X*^1^, …, *X*^*n*^ are min-parallel and some are max-parallel. However, if they are all (pairwise) min-parallel or all max-parallel, we have essentially the same situation as with a simple parallel arrangement of *n* durations.

**Lemma 3.3**. *If X*^1^, …, *X*^*n*^ *are all min-parallel or max-parallel in an* SP *composition, this composition can be presented as T* = *A*^1^ ∧ … ∧ *A*^*n*^ *or T* = *A*^1^ ∨ … ∨ *A*^*n*^, *respectively. In either case*, (*A*^1^, …, *A*^*n*^) ↫ (λ^1^, …, λ^*n*^) *and the Prolongation Assumption holds for any R* = *r*.

*Proof*. For the min-parallel case, by a minor modification of the proof of Lemma 2.6 we present the SP composition as

$$\underset{=A^{1}}{\underset{︸}{{\text{SP}}^{1}(X^{1},\ldots )}}\wedge {\text{SP}}^{2}(X^{2},\ldots ,X^{n},\ldots ),$$

or

$$\begin{array}{l} \;\left({\text{SP}}^{1}(X^{1},\ldots )\wedge {\text{SP}}^{2}(X^{2},\ldots ,X^{n},\ldots )+X\right)\wedge Y\\ =\underset{=A^{1}}{\underset{︸}{\left({\text{SP}}^{1}(X^{1},\ldots )+X\right)}}\wedge \left({\text{SP}}^{2}(X^{2},\ldots ,X^{n},\ldots )+X\right)\wedge Y, \end{array}$$

or else

$$\begin{array}{l} \left({\text{SP}}^{1}(X^{1},\ldots )\wedge {\text{SP}}^{2}(X^{2},\ldots ,X^{n},\ldots )\wedge X\right)+Y\\ =\underset{=A^{1}}{\underset{︸}{\left({\text{SP}}^{1}(X^{1},\ldots )+Y\right)}}\wedge \left({\text{SP}}^{2}(X^{2},\ldots ,X^{n},\ldots )\wedge X+Y\right). \end{array}$$

Then we analogously decompose SP^2^(*X*^2^, …, *X*^*n*^, …) achieving *A*^1^ ∧ *A*^2^ ∧ SP^3^(*X*^3^, …, *X*^*n*^, …), and proceed in this fashion until we reach the required *A*^1^ ∧ … ∧ *A*^*n*^. The pattern of selective influences is seen immediately, and the Prolongation Assumption follows by the monotonicity of the SP compositions. The proof for the max-parallel case is analogous.                       □

**Theorem 3.4**. *If X*^1^, …, *X*^*n*^ *are min-parallel in an* SP *composition, then for any r*, *t*, *c*^(*n*)^(*t*) ≤ 0 *if n is even and c*^(*n*)^(*t*) ≥ 0 *if n is odd. Consequently, for any t*, *C*^(*n*)^(*t*) ≤ 0 *if n is even and C*^(*n*)^(*t*) ≥ 0 *if n is odd. If X*^1^, …, *X*^*n*^ *are max-parallel, then for any r*, *t*, *c*^(*n*)^(*t*) ≥ 0, *and for any t*, *C*^(*n*)^(*t*) ≥ 0.

*Proof*. Follows from Lemma 3.3 and Theorems 3.1 and 3.2.                       □

### 3.2. Sequential processes

#### 3.2.1. Simple serial architectures of size *n*

**Theorem 3.5**. *If T* = *X*^1^ + … + *X*^*n*^, *then for any r*, *t*, *c*^[*n*]^(0, *t*) ≥ 0, *while c*^[*n*]^(*t*, ∞) ≤ 0 *if n is even and c*^[*n*]^(*t*, ∞) ≥ 0 *if n is odd; moreover, c*^[*n*]^(0, ∞) = 0 *for any r. Consequently, for any t*, *C*^[*n*]^(0, *t*) ≥ 0, *while C*^[*n*]^(*t*, ∞) ≤ 0 *if n is even and C*^[*n*]^(*t*, ∞) ≥ 0 *if n is odd; moreover, C*^[*n*]^(0, ∞) = 0.

*Proof*. By induction on *n*, the case *n* = 1 being true by the Prolongation Assumption:

$$c^{\left[1\right]}\left(0,t\right)=c^{\left[1\right]}\left(t,\infty \right)=h_{1}\left(t\right)-h_{2}\left(t\right)\geq 0,$$

and

$$\underset{t\to \infty }{\lim}c^{\left[1\right]}\left(0,t\right)=\underset{t\to 0}{\lim}c^{\left[1\right]}\left(t,\infty \right)=0.$$

Let the statement of the theorem hold for all natural numbers up to and including *n* − 1 ≥ 1. Using the recursive representations (59)–(60),

(63) $$\begin{array}{l} c^{\left[n\right]}\left(0,t\right)=\int_{0}^{t}c_{i_{w}=1}^{\left[n-1\right]}\left(0,\tau \right)d\tau -\int_{0}^{t}c_{i_{w}=2}^{\left[n-1\right]}\left(0,\tau \right)d\tau \\ =\int_{0}^{t-x_{1}^{w}}c^{\left[n-1\right]}\left(0,\tau \right)d\tau -\int_{0}^{t-x_{2}^{w}}c^{\left[n-1\right]}\left(0,\tau \right)d\tau \;,\\ =\int_{t-x_{2}^{w}}^{t-x_{1}^{w}}c^{\left[n-1\right]}\left(0,\tau \right)d\tau \end{array}$$

which is ≥ 0 since *c*^[*n*−1]^(0, τ) ≥ 0 and *t* − *x*^*w*^_2_ ≤ *t* − *x*^*w*^_1_. Analogously,

(64) $$\begin{array}{l} c^{\left[n\right]}\left(t,\infty \right)=\int_{t}^{\infty }c_{i_{w}=1}^{\left[n-1\right]}\left(\tau ,\infty \right)d\tau -\int_{t}^{\infty }c_{i_{w}=2}^{\left[n-1\right]}\left(\tau ,\infty \right)d\tau \\ =\int_{t-x_{1}^{w}}^{\infty }c^{\left[n-1\right]}\left(\tau ,\infty \right)d\tau -\int_{t-x_{2}^{w}}^{\infty }c^{\left[n-1\right]}\left(\tau ,\infty \right)d\tau \;,\\ =-\int_{t-x_{2}^{w}}^{t-x_{1}^{w}}c^{\left[n-1\right]}\left(\tau ,\infty \right)d\tau \end{array}$$

which is ≤ 0 if *n* is even and ≥ 0 if *n* is odd. Applying the mean value theorem to the results of (63) and (64), we get, for some *t* − *x*^*w*^_2_ < *t*′, *t*″ < *t* − *x*^*w*^_1_

$$\int_{t-x_{2}^{w}}^{t-x_{1}^{w}}c^{\left[n-1\right]}\left(0,\tau \right)d\tau =c^{\left[n-1\right]}\left(0,t^{\prime }\right)\left(-x_{1}^{w}+x_{2}^{w}\right),$$

$$\int_{t-x_{2}^{w}}^{t-x_{1}^{w}}c^{\left[n-1\right]}\left(\tau ,\infty \right)d\tau =c^{\left[n-1\right]}\left(t^{\prime \prime },\infty \right)\left(-x_{1}^{w}+x_{2}^{w}\right),$$

and, as *c*^[*n*−1]^(0, ∞) = 0, both expressions tend to zero as, respectively, *t* → ∞ (implying *t*′ → ∞) and *t* → 0 (implying *t*″ → 0).                       □

#### 3.2.2. Multiple sequential processes in arbitrary SP networks

In a composition SP(*X*^1^, …, *X*^*n*^, …), the components *X*^1^, …, *X*^*n*^ are considered sequential if any two of them are sequential. By analogy with Theorem 2.9 for two sequential processes and with Theorem 3.4 for parallel *X*^1^, …, *X*^*n*^, one might expect that the result for the simple sequential arrangement *X*^1^ + … + *X*^*n*^ will also extend to *n* sequential components of more complex compositions SP(*X*^1^, …, *X*^*n*^, …). However, this is not the case, as one can see from the following counterexample.

Consider the composition

(65) $$\text{SP}(X^{1},X^{2},X^{3},Y)=\left(X^{1}+X^{2}+X^{3}\right)\wedge \left(Y=2\right),$$

with (*X*^1^, *X*^2^, *X*^3^) selectively influenced by binary factors, so that

(66) $$\begin{array}{l} x_{1}^{1}=x_{1}^{2}=x_{1}^{3}=0,\\ x_{2}^{1}=x_{2}^{2}=x_{2}^{3}=1. \end{array}$$

It follows that

(67) $$\begin{array}{l} t_{111}=0,\\ t_{112}=t_{121}=t_{211}=1,\\ t_{122}=t_{212}=t_{221}=t_{222}=2. \end{array}$$

This is clearly a sequential arrangement of the three durations *X*^1^, *X*^2^, *X*^3^, but one can easily check that *c*^[3]^(0, *t*) here is not nonnegative for all *t*. For instance, at *t* = 3 we have, by direct computation, *c*^[3]^(0, *t*) = −1. We conclude that there is no straightforward generalization of Theorems 3.5 to arbitrary SP compositions.

## 4. Conclusion

The work presented in this paper is summarized in the abstract. By proving and generalizing most of the known results on the interaction contrast of distribution functions, we have demonstrated a new way of dealing with SP mental architectures. It is based on conditioning all hypothetical components of a network on a fixed value of a common source of randomness *R* (the “hidden variable” of the contextuality analysis in quantum theory), which renders these components deterministic quantities, and then treating these deterministic quantities as random variables with shifted Heaviside distribution functions.

The potential advantage of this method can be seen in the fact that the shifted Heaviside functions have the simplest possible arithmetic among distribution functions: for every time moment it only involves 0's and 1's. As a result, the complexity of this arithmetic does not increase with nonlinearity of the relations involved. Thus, Dzhafarov and Schweickert (1995); Cortese and Dzhafarov (1996), and Dzhafarov and Cortese (1996) argued that composition rules for mental architectures need not be confined to +, ∧, ∨. They analyzed architectures involving other associative and commutative operations, such as multiplication. Due to mathematical complexity, however, this work was confined to networks consisting of two components that are either stochastically independent or monotone functions of each other. It remains to be seen whether the approach presented here, *mutatis mutandis*, will lead to significant generalizations in this line of work.

The limitations of the approach, however, are already apparent. Thus, we were not able to achieve any progress over known results in applying it to Wheatstone bridges (Schweickert and Giorgini, 1999; Dzhafarov et al., 2004). The possibility that the “architecture” (composition rule) itself changes as one changes experimental factors makes the perspective of a general theory based on our approach even more problematic (e.g., Townsend and Fific, 2004). It seems, however, that these problems are not specific for just our approach.

### Conflict of interest statement

The authors declare that the research was conducted in the absence of any commercial or financial relationships that could be construed as a potential conflict of interest.
