# Corrigendum: Noncontextuality with marginal selectivity in reconstructing mental architectures

**DOI:** 10.3389/fpsyg.2016.00437

**Published:** 2016-03-30

**Authors:** Ru Zhang, Ehtibar N. Dzhafarov

**Affiliations:** Department of Psychological Sciences, Purdue UniversityWest Lafayette, IN, USA

**Keywords:** interaction contrast, mental architectures, response time, series-parallel networks, corrigendum

This corrigendum note points out and corrects two mistakes found in the paper cited in the title. These mistakes do not affect the correctness of the statements proved and expressions derived.

1. In Zhang and Dzhafarov (2015), Lemma 2.6 (p. 7) and Lemma 3.3 (p. 10) are formulated for series-parallel (SP) architectures in which the minimum (∧) and maximum (∨) operations may be intermixed. The proofs are shown for the min-parallel arrangement of the selectively influenced processes, with the correct statement that the max-parallel arrangement is dealt with analogously. However, by an oversight, the proof for the min-parallel arrangement is shown only for homogeneous SP_∧_ architectures, those that cannot contain ∨ operations. The statements of the lemmas are correct despite this oversight, because the proofs remain valid if the rightmost ∧*Y* and ∧*X* in all expressions of the form
  $$\left({\text{SP}}^{1}(\ldots )\wedge {\text{SP}}^{2}(\ldots )+X\right)\underset{↑}{\wedge }Y\text{ and}\left({\text{SP}}^{1}(\ldots )\wedge {\text{SP}}^{2}(\ldots )\underset{↑}{\wedge }X\right)+Y$$
  are replaced with ∨*Y* and ∨*X*, respectively.
2. Equations (61) and (62) on p. 9 should be disregarded: one of them, (62), contains typos, and both are shown in the wrong place. These transformations are only valid in the context of Theorem 3.5, for sequential architectures, and this is the only place where they are used, in Equations (63) and (64) on p. 11.

(A typo: in Equation (60) on p. 9, *c*^[*n*]^(*t*, ∞) should be $c_{r}^{\left[n\right]}\left(t,\infty \right)$.)

## Author contributions

All authors listed, have made substantial, direct and intellectual contribution to the work, and approved it for publication.

### Conflict of interest statement

The authors declare that the research was conducted in the absence of any commercial or financial relationships that could be construed as a potential conflict of interest.
